# Development of an enterocutaneous fistula from an intestinal perforation in a patient with autosomal dominant polycystic kidney disease

**DOI:** 10.1007/s13730-022-00716-z

**Published:** 2022-07-05

**Authors:** Yuki Nakayama, Naoki Sawa, Tatsuya Suwabe, Akinari Sekine, Masayuki Yamanouchi, Daisuke Ikuma, Hiroki Mizuno, Yuki Oba, Eiko Hasegawa, Junichi Hoshino, Shuichiro Matoba, Yoshifumi Ubara

**Affiliations:** 1 Nephrology Center, Toranomon Hospital Kajigaya, 1-3-1, Kajigaya, Takatsu, Kawasaki, Kanagawa 213-8587 Japan; 2 Department of Gastrointestinal Surgery, Toranomon Hosipital, Tokyo, Japan; 3 Okinaka Memorial Institute for Medical Research, Toranomon Hospital, Tokyo, Japan

**Keywords:** Autosomal dominant polycystic kidney disease (ADPKD), Intestinal perforation, Enterocutaneous fistula, Transcatheter arterial embolization (TAE)

## Abstract

We herein report a case of enterocutaneous fistula in a patient with autosomal dominant polycystic kidney disease (ADPKD). A 37-year-old Japanese man was admitted to our hospital. Three months prior to transfer to our hospital, he developed intense flank pain with gross hematuria. His serum creatinine had decreased to 7.8 mg/dL and hemodialysis was started, but gross hematuria persisted and he developed hypotension. Upon admission, plain chest radiography did not reveal any free air, but computed tomography (CT) showed generalized ventral subcutaneous air from the head to the lower extremities and enlarged kidneys. Enterography showed leakage of contrast medium from the descending colon into the subcutaneous area. C-reactive protein was 23.1 mg/dL. A colostomy was placed in the transverse colon proximal to the perforation, and systemic subcutaneous drainage was performed. The fever subsequently resolved, and the C-reactive protein test became negative. Three months later, renal artery embolization was performed, and 12 months thereafter, CT showed a marked decrease in kidney size. We assume that a markedly enlarged kidney leaded to intestinal perforation, which developed into an enterocutaneous fistula. Consequently, intestinal fluid leaked into the subcutaneous cavity of the abdominal wall and spread systemically, resulting in extensive subcutaneous abscesses.

## Introduction

Autosomal dominant polycystic kidney disease (ADPKD) is characterized by an increase in kidney size that is inversely proportional to the deterioration of kidney function. The disease has both renal and extra-renal manifestations [1]. The latter include gastrointestinal complications; for example, colonic diverticulosis has been reported to be common [2]. Another gastrointestinal complication is ileus, which is caused by compression of the gastrointestinal tract due to enlargement of the liver and kidneys [3]. Here, we report a case of ADPKD with a markedly enlarged kidney, whose intestinal perforation led to an enterocutaneous fistula of the abdominal wall, allowing intestinal fluid to spread systemically.

## Case report

**Fig. 1:**
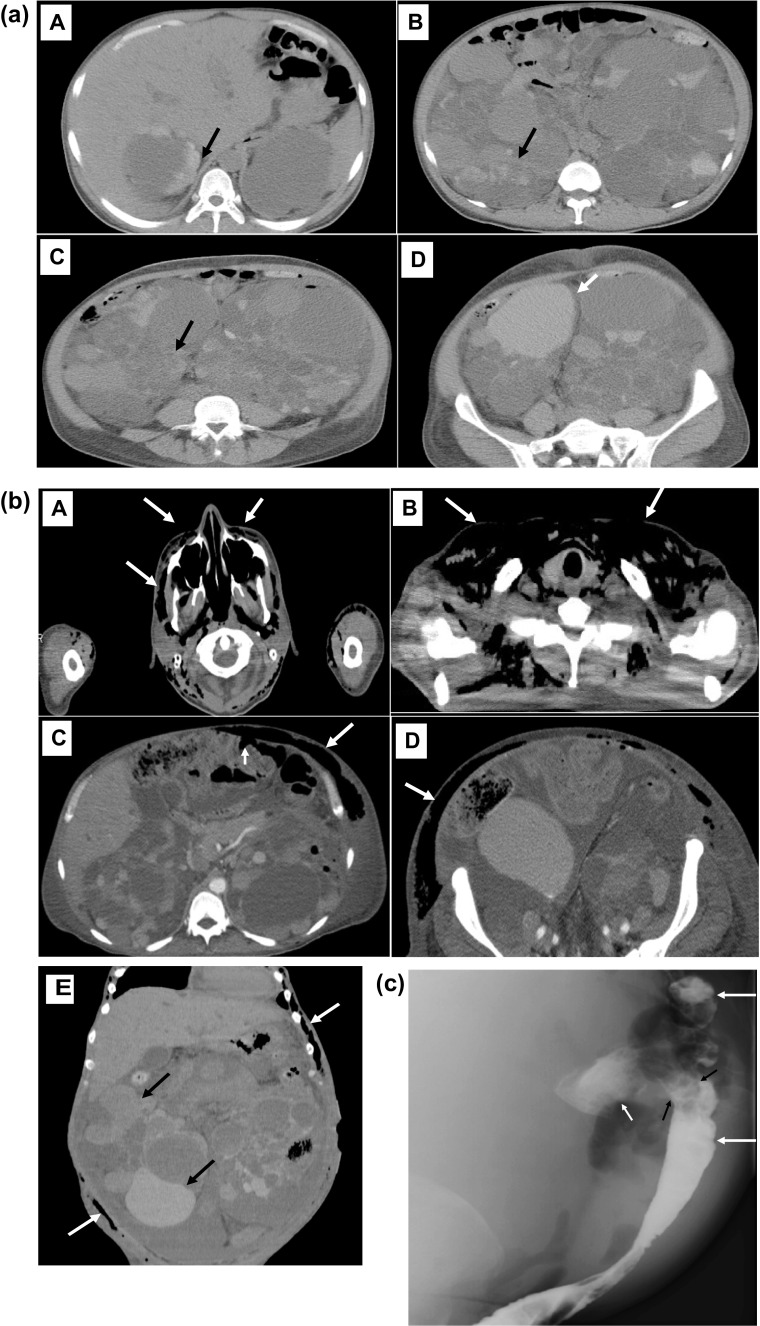

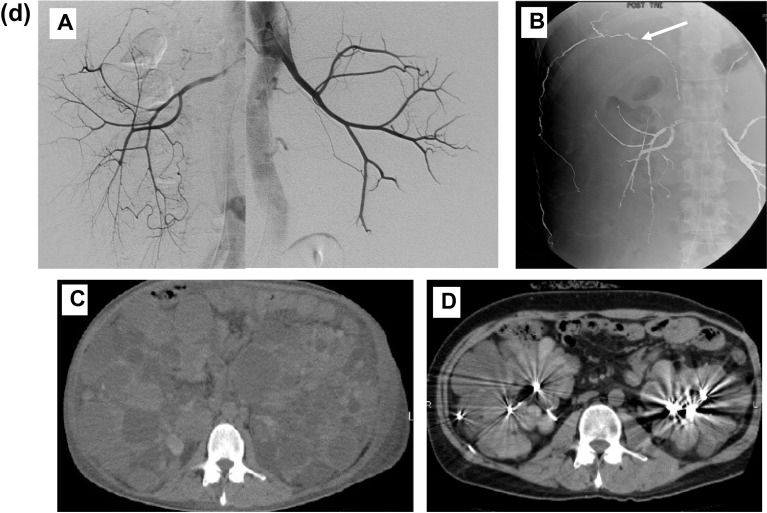
**a** Computed tomography (CT) 3 months before transfer to our hospital showed many areas suggestive of active renal hemorrhage (high density area with irregular border, black arrow) or old hemorrhage (higher density area, white arrow) in both kidneys. **b** Computed tomography showed generalized ventral subcutaneous free air including pus-filled fecal discharge (arrow) from the head to the lower abdomen. A, head; B, neck, C; upper abdomen (small arrow indicates the cystic (suggestive of diverticulum) fistula between the descending colon and the subcutaneous area); D, lower abdomen; E, coronary image of CT shows repeated cyst hemorrhage (high densitry area, black arrow) and subcutaneous free air including pus-filled fecal discharge (white arrow). **c** Enterography shows leakage of contrast medium from the descending colon (large arrow) into the subcutaneous area (small arrow). The perforation site was dilated and appeared to correspond to the diverticulum (black arrow). **d** Results of imaging and procedures. A, Renal artery angiography shows well-developed and stretched renal artery branches. B, Renal artery embolization was performed with a microcoil (arrow). C, Computed tomography before renal artery embolization. D, Computed tomography 12 months after renal artery embolization

A 37-year-old Japanese man was admitted to our hospital for examination of fever and hypotension. He had been diagnosed with ADPKD because of hypertension (BP170/95 mmHg) and hematuria at the age of 26. His father had the same disease. Thereafter mild abdominal pain and hematuria were present but temporary. Three months prior to transfer to our hospital, he developed intense flank pain with gross hematuria. Compared to one month ago, his serum creatine decreased to from 5.0 mg/dL to 7.8 mg/dL and serum hemoglobin decreased from 9.0 g/dL to 6.0 g/dL. Urinalysis showed urine sediment contained 100 > red cells and 5–10 white cells per high-power field. Computed tomography (CT) showed many areas suggestive of active renal hemorrhage or old hemorrhage in both kidneys (total kidney volume of 6606 cm^3^; right kidney of 10.8 × 20.6 × 22.7 cm and left kidney of 18.5 × 17.2 × 23.8 cm) (Fig. 1a). Gross hematuria persisted and red blood cell transfusions was repeated, and hemodialysis was initiated as further conservative management was considered difficult. Then, he was transferred to our hospital a month after the start of hemodialysis because gross hematuria persisted, and antibiotic-resistant fever and hypotension developed.

On admission, the patient was 176 cm tall, and his dry weight was 86 kg. His blood pressure was 94/54 mmHg, and his body temperature, 38.5 ℃. Heart and breath sounds were normal. The abdomen was markedly distended and elastic hard. The entire abdomen was tender, and the abdominal skin was red. The maximum circumference of the abdomen was 113 cm. Generalized edema was observed.

The complete blood count was as follows: erythrocytes, 2.20 × 10^6^/μL; hemoglobin, 6.1 g/dL; hematocrit, 19.3%; leucocytes, 9800/μL; and thrombocytes, 30.1 × 10^4^/μL. Blood chemistry tests found a total protein of 6.5 g/dL; albumin, 1.3 g/dL; urea nitrogen, 76 mg/dL; creatinine, 5.3 mg/dL; and uric acid, 8.2 mg/dL. C-reactive protein (CRP) was 23.1 mg/dL, and the erythrocyte sedimentation rate was greater than 110 mm/h. Blood culture test could not identify any significant causative organisms.

Plain chest radiography did not reveal any free air, but CT showed generalized ventral subcutaneous air from the head to the lower extremities (Fig. 1b). CT also showed enlarged polycystic kidneys (total kidney volume of 8273 cm^3^; right kidney of 12.2 × 21.7 × 25.6 cm and left kidney of 19.0 × 18.5 × 25.7 cm). Hepatic cysts were also detected, but hepatomegaly was mild. A test puncture of the subcutaneous air revealed pus-filled fecal discharge. When enterography was performed from the anus, contrast medium was seen to leak from the descending colon into extra-colon space including the subcutaneous area of the abdominal wall. The perforation site was dilated and appeared to correspond to a diverticulum (Fig. 1c). A colostomy was placed in the transverse colon proximal to the perforation area, and the perforation area was left untreated surgically. Systemic subcutaneous drainage was performed for subcutaneous emphysema sites. Even after transfer to our hospital, the renal hemorrhage continued, and anemia was treated with repeated red blood cells transfusions. In the third month of hospitalization when the fever resolved, and the CRP test was confirmed negative, renal artery embolization was performed with a microcoil, as described previously [4]. Twelve months thereafter, CT revealed a marked decrease in the size of the kidneys (total kidney volume of 2446 cm^3^; right kidney of 12.0 × 11.3 × 22.1 cm; left kidney, 8.9 × 9.1 × 20.8 cm) (Fig. 1d). Eight years after the discharge from our hospital when I am writing this paper, the patient can eat normally and is doing well.

## Discussion

In patients with ADPKD, intestinal compression due to enlarged liver and kidneys has been reported to cause gastrointestinal symptoms, including upper digestive tract symptoms, such as heartburn, nausea, vomiting, loss of appetite, and early satiety; severe constipation; and odd-shaped feces [1]. Kato et al. reported a case of a giant cystic kidney in which perforated intestine protruded out of the abdominal wall because of thinning of the abdominal wall after the onset of paralytic ileus of the small intestine [3]. Several papers reported that intestinal perforation was common in patients with cystic kidneys and hypothesized that the diverticulum was the cause; however, none of the reported studies could prove that the diverticulum was involved at the site of the perforation. Domínguez Fernández et al. investigated whether sigmoid perforation due to diverticulitis can occur in the postoperative course of allogenic kidney transplantation: patients with ADPKD did not find a higher prevalence of diverticulosis at the site of colonic perforation, although sigmoid perforation was seen [5]. Scotti et al. investigated 717 patients who underwent kidney transplant between 2000 and 2010 and also found that patients with ADPKD more frequently developed colonic diverticulosis and colonic perforation [6]. They suggested that colonic perforation might be caused by renal cyst infection, but they could not prove that it involved the diverticulum [6]. Duarte-Chavez et al. found that colonic diverticulosis, diverticulitis, and diverticular bleeding are considerably more common in patients with ADPKD than in the general population and that colonic surgery was less prevalent in those patients with diverticulitis [7]. The group found higher odds ratios for diverticular disease in patients with non-steroidal anti-inflammatory drug use, hypertension, constipation, and ADPKD, but did not find a relation between intestinal perforation and diverticulosis [7]. In summary, no specific data have been reported on the relationship between intestinal diverticulosis and intestinal perforation. However, with the addition of the specific renal enlargement and associated increased intra-abdominal pressure in patients with ADPKD, it seemed significant that intestinal diverticulosis could lead to intestinal perforation in patients with ADPKD.

Cyst hemorrhage is a common cause of gross hematuria, and cyst hemorrhage event is a risk factor for renal prognosis in ADPKD. There is a question as to whether cyst hemorrhage really causes renal size enlargement. The report by Ubara et al. provides one answer to this question. He reported on TAE for ADPKD in a review article. In the first case, renal hemorrhage led to renal enlargement, and TAE was performed to treat hemostasis, resulting not only in hemostasis but also in a reduction in the size of the kidney [1]. Indeed, the incidence of hematuria and renal cyst hemorrhage was suppressed in tolvaptan group compared to placebo group in TEMPO 3:4 trial. The fact that the use of tolvaptan also reduced the rate of increase in renal size and renal hemorrhage may suggest a relationship between renal hemorrhage and increased renal size (8).

From the clinical course of our case, we can assume that the following events occurred. First, repeated renal cyst hemorrhage, as evidenced by gross hematuria, caused the kidneys to an increase in size, and the resulting increase in intra-abdominal pressure caused an intestinal perforation. The intestinal perforation leads usually to leakage of intestinal contents into the abdominal cavity, resulting in peritonitis. In our case, it developed into an enterocutaneous fistula beyond the narrow abdominal cavity because the pressure associated with renal enlargement was too high, so intestinal contents moved into the subcutaneous cavity of the abdominal wall and then spread systemically, resulting in extensive subcutaneous abscess.

The total kidney volume 3 months earlier was 6606 cm^3^, and the total kidney volume at the time of transfer to our hospital was 8273 cm^3^, clearly indicating an increase in kidney size. Twelve months after renal artery embolization, the total kidney volume was decreased to 2446 cm^3^. These data indicate that repeated renal hemorrhage causes a rapid increase in renal size, and that stopping the renal hemorrhage by renal embolization causes a rapid reduction in renal size.

In conclusion, we experienced a patient of ADPKD with a markedly enlarged kidney. The intestinal perforation developed into an enterocutaneous fistula, through which intestinal contents spread systemically. Renal arterial embolization therapy performed after clearance of the infection reduced the size of the enlarged kidneys and lowered the intra-abdominal pressure, preventing recurrence of the enterocutaneous fistula.

## Data Availability

All data generated or analyzed during this study are included in this article. Further inquiries can be directed to the corresponding author.
